# Supplementary material for: Cycad phylogeny predicts host plant use of Eumaeus butterflies
Source: Ecol Evol. 2023 Apr 10;13(4):e9978. doi: 10.1002/ece3.9978 (PMC10085819; doi:10.1002/ece3.9978)
Supplement: Supplementary file 1 — Data S1 [file ECE3-13-e9978-s001.pdf]

Cycad phylogeny predicts host plant use of *Eumaeus* butterflies

Laura Sierra-Botero[1*], Michael Calonje[2], Robert K. Robbins[3], Neil Rosser[4], Naomi E. Pierce[4,5], Cristina López-Gallego[1†], Wendy A. Valencia-Montoya[4,5†*]

[1]**Instituto de Biología, Universidad de Antioquia, Medellín, Antioquia, Colombia**

[2]**Montgomery Botanical Center, Coral Gables, Florida 33156, USA**

[3]Department of Entomology, Smithsonian Institution, Washington, DC 20013-7012, USA

[4]**Department of Organismic and Evolutionary Biology, Harvard University, Cambridge, MA, USA**

[5]**Museum of Comparative Zoology, Harvard University, Cambridge, MA, USA**
†supervised this work

[*]**Authors for correspondence: Laura Sierra-Botero and Wendy A. Valencia-Montoya**
e-mails: lrrsrr@gmail.com and wvalenciamontoya@g.harvard.edu

Supplementary information

1. *Eumaeus* host-plant records and distribution

Table 1. Records of larval host plant use by *Eumaeus* species.“Observed in the field” refer to direct observations of larvae feeding on *Zamia* leaves derived from fieldwork conducted in this study. “Literature report” is based on published papers. All reports are for plants in their native distribution ranges.

Eumaeus	Host plant	Locality	Source	Host-plant report
E. toxana	*Z. boliviana*	Federico Román, BO-PA, Bolivia	iNaturalist obs 31700705	Inferred from coordinates by Michael Calonje
E. toxana	*Z. boliviana*	La Junta, Bolivia	iNaturalist obs 57490049	Inferred from coordinates by Michael Calonje
E. toxana	*Z. cupatiensis*	El Varillal, Amazonas, Colombia	Sierra-Botero, Valencia-Montoya, López-Gallego, 2022 /CEUA 31501-31504	Observed in the field
E. toxana	pr. *Z. lecontei* /*Z. amazonum*	Cerro de la Neblica Baseca, Amazonas, Venezuela	Robbins et al., 2021	Literature report. Inferred from coordinates by Michael Calonje
E. toxana	pr. *Z. macrochiera* /*Z. ulei*	Rio Sucusari, Loreto, Peru	Robbins et al., 2021	Literature report. Inferred from coordinates by Michael Calonje
E. toxana	pr. *Z. amazonum* /*Z. poeppgiana*	Morona-Santiago, Ecuador	Robbins et al., 2021	Literature report. Inferred from coordinates by Michael Calonje
E. toxana	*Z. ulei*	ARCC, Las Piedras River, Peru /Madre de Dios River, Madre de Dios, Peru	iNaturalist obs 105616726	Inferred from coordinates by Michael Calonje

E. toxana	*Z. sp inédita*	Paucartambo Province, Peru	iNaturalist obs 103468988	Inferred from coordinates by Michael Calonje
E. toxana	*Z. poeppigiana*	San Martín, Peru	iNaturalist obs 105690568	Inferred from coordinates by Michael Calonje
E. toxana	*Z. amazonum, Z. ulei,* or *'Z. jirijirimensis'* (now accepted as *Z. lecontei*)	Cerro Morroco, Pacoa,Vaupés, Colombia	ColombiaBio:SINCHI:Apaporis:MARIPOSAS:2018:194	Inferred from coordinates by Michael Calonje
E. minyas	*Z. tolimensis*	Tolima, Colombia	Castro, 2019 CBUCES 4702	Observed in the field
E. minyas	*Z. incognita*	Río Claro, Antioquia, Colombia	MEFLG sin código S. A. Ramírez 1994	Inferred from coordinates by Cristina López-Gallego
E. minyas	*Z. incognita*	Puerto Berrío, Antioquia, Colombia	iNaturalist obs 34094890	Inferred from coordinates by Michael Calonje
*E. minyas**	*Z. incognita*	Maceo, Antioquia, Colombia	Sierra-Botero, Valencia-Montoya, López-Gallego, 2021 /Reported in Valencia-Montoya et al., 2017 as *E. godartii*	Observed in the field
E. minyas	*Z. boliviana*	Mato Grosso, Brasil	Segalla et al., 2021	Literature report
E. godartii	*Z. obliqua /Z. manicata*	Sapzurro, Chocó, Colombia	MEFLG 14828	Inferred from coordinates by Cristina López-Gallego
E. minyas	*Z. encephalarthoides*	Cañón del Chicamocha, Colombia	González, 2004	Literature report
E. minyas	*Z. encephalarthoides*	San Gil, Cimitarra, Girón, Santander, Colombia	iNaturalist obs 29387964<br>iNaturalist obs 31433997<br>iNaturalist obs 75953118<br>CEUA 12215<br>ICN 054595	Inferred from coordinates by Michael Calonje
E. minyas	*Z. encephalarthoides*	Vereda La mina, Puerto Nare, Colombia	CEUA 26336	Inferred from coordinates by Michael Calonje
E. minyas	*Z. hymenophyllidia*	Puerto Nariño, Amazonas, Colombia	ICN 089609	Inferred from coordinates by Michael Calonje
E. minyas	*Zamia aff. muricata*	San Juan de Arama Rio Güejar, Meta, Colombia	IAvH-E-9622	Inferred from coordinates by Michael Calonje
E. minyas	*pr. Z. incognita /Z. imbricata*	Victoria, Caldas, Colombia	IAvH-E-9624	Inferred from coordinates by Michael Calonje
E. minyas	*pr. Z. poeppigiana/ Z. ulei / Z. urep*	Padre Abad, Peru	iNaturalist obs 91195200	Inferred from coordinates by Michael Calonje
E. minyas	*Z. ulei*	Tambopata, Peru	iNaturalist obs 68764827	Inferred from coordinates by

				Michael Calonje
E. minyas	*Z. ulei*	Entre Alegría y Planchón, Peru	iNaturalist obs 34613717	Inferred from coordinates by Michael Calonje
E. minyas	*Z. ulei*	Tahuamanu Province, Peru	iNaturalist obs 11572166	Inferred from coordinates by Michael Calonje
E. minyas	*Z. poeppigiana*	San Martín, Peru	iNaturalist obs 39751148	Inferred from coordinates by Michael Calonje
E. minyas	*Z. poeppigiana*	San Martín, Peru	iNaturalist obs 9811541	Inferred from coordinates by Michael Calonje
E. minyas	*Cycas revoluta*	Manu, Peru	iNaturalist obs 20415254	Inferred from coordinates by Michael Calonje
E. godartii	*Z. amplifolia*	Bajo Calima, Valle del Cauca, Colombia	Sierra-Botero, López-Gallego, Valencia-Montoya, 2021	Observed in the field
E. godartii	*Z. chigua*	Buenaventura, Valle, Colombia	Sierra-Botero, López-Gallego, Valencia-Montoya, 2021	Observed in the field
E. godartii	*Z. sp inédita*	Buenaventura, Valle, Colombia	Sierra-Botero, López-Gallego, Valencia-Montoya, 2021	Observed in the field
E. godartii	*Z. obliqua*	Nuquí, Colombia	CBUCES Valencia-Montoya, 2020	Observed in the field
E. godartii	*Z. obliqua*	Bahia Solano, Los Katíos, Chocó, Colombia	iNaturalist obs 70523609 iNaturalist obs 20075063	Inferred from coordinates by Michael Calonje
E. godartii	*Z. obliqua /Z. manicata*	Sapzurro, Chocó, Colombia	MEFLG 14828	Inferred from coordinates by Cristina López-Gallego
E. godartii	*Z. manicata*	Mutatá, Antioquia, Colombia	iNaturalist obs 25039196	Inferred from coordinates by Cristina López-Gallego
E. godartii	*Z. manicata*	Turbo, Antioquia, Colombia	MEFLG sin código Gallego, 1942	Inferred from coordinates by Cristina López-Gallego
E. godartii	*Z. manicata*	Parque Natural Katíos, Riosucio, Chocó, Colombia	IAvH-E-9627	Inferred from coordinates by Cristina López-Gallego
E. godartii	*Z. manicata*	Darién, Panama	Santos Murgas & Abrego, 2016	Literature report
E. godartii	*Z. acuminata*	El Rodeo, Costa Rica	Cascante Marín & Vega Araya, 2012	Literature report
E. godartii	*Z. fairchildiana*	Costa Rica	Jones, 2002 in Cascante Marín & Vega Araya, 2012 /P. DeVries curated by Robbins et al., 2021 /López-Gallego, 2007	Literature report
E. godartii	*Z. stevensonii*	Chagres Valley, Panamá	Prado et al., 2014 curated by Robbins et al., 2022	Literature report

E. godartii	*Z. neurophyllidia*	Costa Rica	DeVries, 1977; Robbins et al., 2021	Literature report
E. godartii	*Z. neurophyllidia**	La Selva, Costa Rica	Clark & Clark, 1991 reported as *Z. skinnerii*	Literature report, curated by Michael Calonje
E. godartii	*Z. ipetiensis*	Kuna Yala, Panamá	Calonje, M. Julio 12, 2007, associated with specimen Calonje MAC07-05 (PMA)	Observed in the field
E. godartii	*Z. lindenii*	Santo Domingo De Los Colorados, Ecuador	iNaturalist obs 103978588 iNaturalist obs 102537073 iNaturalist obs 102552998 iNaturalist obs 93677384	Inferred from coordinates by Michael Calonje
E. godartii	*Z. obliqua/Z. chigua*	El cantón de San Pablo, Chocó, Colombia	iNaturalist obs 10722844	Inferred from coordinates by Cristina López-Gallego
E. godartii	*Z. lindleyi*	Chiriquí province, Panamá	Taylor Blake. et al., 2012	Literature report
E. godartii	*Z. nesophila*	Island of northwest Panama	Taylor Blake., 2020	Literature report
E. godartii	*Z. cunaria*	Island of north central Panama, near Caribbean coast, Panama	Taylor Blake., 2020	Literature report
E. godartii	*Z. nana*	West central Panama	Taylor Blake., 2020	Literature report
E. godartii	*Z. dressleri*	Colón, Kuna Yala, Panama	Taylor Blake., 2020	Literature report
E. godartii	*Z. pseudomonticola*	Chiriquí, Panama	Taylor Blake., 2020	Literature report
E. godartii	*Z. elegantissima*	Colón province, Panamá	Taylor Blake. et al., 2012	Literature report
E. childrenae	*Ceratozamia mexicana*	Huasteca, México	Contreras-Medina et al., 2003	Literature report
E. childrenae	*Ceratozamia chimalapensis*	Veracruz, México	Ramírez-Restrepo et al., 2017	Literature report
E. childrenae	*Cycas revoluta*	Veracruz, México	Murillo, 2002 curated by Robbins et al., 2021	Literature report
E. childrenae	*Dioon edule*	Veracruz /Taumalipas /Nueva León, México	Murillo, 2002 curated by Robbins et al., 2021	Literature report
E. childrenae	*Dioon purpusii*	Nuevo León, México	O. Kendall & C. A. Kendall curated by Robbins et al., 2021	Literature report
E. childrenae	*Dioon holmgrenii*	Oaxaca, México	Ruiz–García, 2020	Literature report
E. childrenae	*Z. cremnophila*	Tabasco, México	Jiménez-Pérez et al., 2017	Literature report
E. childrenae	*Z. vasquezii**	Reared in San Antonio, Texas - perhaps intercepted at a border crossing,	Robbins et al., 2021, reported as *Zamia probably fischeri* Miq.	Literature report, curated by Michael Calonje

		México		
E. childrenae	*Z. furfuracea* Ait.	Veracruz, México	Robbins et al., 2021	Literature report
E. childrenae	*Z. fischeri**	San Luis Potosí, México	Robbins et al., 2021 reported as *Z. furfuracea*	Literature report, curated by Michael Calonje
E. childrenae	*Z. fischeri*	Huasteca /Hidalgo and Queretaro, México	Contreras-Medina et al., 2003	Literature report
E. childrenae	*Macrozamia*	Veracruz, México	Murillo, 2002 curated by Robbins et al., 2021	Literature report
E. toxea	*Z. furfuracea*	Veracruz, México	Martínez-Lendech et al., 2007	Literature report
E. toxea	*Z. paucijuga*	Oaxaca, México	Ruiz-García et al., 2015	Literature report
E. toxea	*Z. loddigesii*	Veracruz, México	Ross, 1964 curated by Robbins et al. 2021; Castillo-Guevara & Rico-Gray, 2002	Literature report
E. atala	*Z. integrifolia* US	Florida, USA; Cayman Islands.	Koi & Daniels, 2015; Robbins et al., 2021; iNaturalist obs 71876257	Literature report
E. atala	*Z. integrifolia* BS	South Abaco, Central Abaco, West Grand, Bahamas	iNaturalist obs 3835311 iNaturalist obs 100647787	Inferred from coordinates by Michael Calonje
E. atala	*Z. pumila*	Everglades National Park, Florida, USA	Tang, 1990	Literature report
E. atala	*Zamia* sp. (yunquilla)	Cuba /Bahamas /Florida, USA.	Robbins et al., 2021	Literature report

Abbreviations: pr, probably; US, United States; BS, Bahamas; *in the original reference reported with a different name, we report the valid name.

2. *Eumaeus* southern clade taxonomy

The specimens of the three species of the southern clade in Colombia were identified and classified using the following definitions of species based on morphological characters from an extensive literature review:

> ***E. toxana*** (Boisduval, 1870): females are the only *Eumaeus* without green/blue scaling on the anterior dorsal wing. Male aedeagus valvae are short, not needle-like. (Robbins et al., 2021). On the dorsal hindwing of both females and males, the green-blue spots are longer and pointier. The longest spot in the center diminishes towards the apex and anal angle (Goodson, 1947). Ventral spots of the hindwing are clotted and pointed, resembling a triangle-like form.

E. minyas (Hübner, 1809): females have reduced green/blue scaling, have a broader wing, and the apex of the forewing is more rounded (Goodson, 1947). Male aedeagus valvae are needle-like (Robbins et al., 2021). All specimens have a thin white line on the margin of the hind and forewings.

E. godartii (Boisduval, 1870): shares with *E. minyas* characters such as green/blue diminishing scaling for females (Goodson, 1947) and the male needle-like genitalia valvae (Robbins et al., 2021). Females have a big, around 1,5 mm apical white spot on the dorsal surface of the forewing (Goodson, 1947) and the same spot (usually smaller) on the ventral side of the forewing. Males do not have the dorsal apical white spot but might have the ventral spot reduced (in comparison to the females).

***E. godartii - E. minyas* intermediate:** green/blue diminishing scaling for females, males with needle-like genitalia valvae. Females have a little, less than 0,8 mm apical white spot on the dorsal surface of the forewing.

To prevent biases coming from potential hybrids or introgressed specimens, we did not include intermediate specimens in the analysis, nor information about their host plants, but their distributions are reported in Fig. 2.

References

Cascante Marín, A., & Vega Araya, G. (2012). El fósil viviente *Zamia acuminata* (Zamiaceae) y la mariposa *Eumaeus godartii* (Lepidoptera: Lycaenidae) en El Rodeo. *Brenesia*, *77*, Article 77.

Castillo-Guevara, C., & Rico-Gray, V. (2002). Is cycasin in *Eumaeus minyas* (Lepidoptera: Lycaenidae) a predator deterrent? *Interciencia*, *27*(9), 465–470.

Clark, D. B., & Clark, D. A. (1991). Herbivores, herbivory, and plant phenology: Patterns and consequences in a tropical rain-forest cycad. In *Plant-animal interactions: Evolutionary ecology in tropical and temperate regions* (Wiley-Interscience, p. 639). Wiley.

Contreras-Medina, R., Ruiz-Jiménez, C. A., & Luna Vega, I. (2003). Caterpillars of *Eumaeus* childrenae (Lepidoptera: Lycaenidae) feeding on two species of cycads (Zamiaceae) in the Huasteca region, Mexico. *Revista De Biologia Tropical*, *51*(1), 201–203.

González, F. (2004). De Colombia , *Zamia encephalartoides* (Zamiaceae) Por Parte De

Eumaeus (Lepidoptera: Lycaenidae). *Revista de La Academia Colombiana de Ciencias Exactas, Fisicas y Naturales*, *XXVIII*, 233–244.

Jiménez-Pérez, N. del C., Moguel-Ordóñez, E. J., Hernández-Jiménez, O. A., & Cruz, M. P. la. (2017). Primer Registro de *Eumaeus childrenae* Sobre la Cícada Microendémica *Zamia cremnophila* (Zamiaceae) en Tabasco, México. *Southwestern Entomologist*, *42*(2), 609–612. https://doi.org/10.3958/059.042.0233

Koi, S., & Daniels, J. (2015). New and revised life history of the Florida hairstreak *Eumaeus atala* (Lepidoptera: Lycaenidae) with notes on its current conservation status. *Florida Entomologist*, *98*(4), 1134–1147. https://doi.org/10.1653/024.098.0418

Martínez-Lendech, N., Córdoba-Aguilar, A., & Serrano-Meneses, M. A. (2007). Body size and fat reserves as possible predictors of male territorial status and contest outcome in the butterfly *Eumaeus toxea* Godart (Lepidoptera: Lycaenidae). *Journal of Ethology*, *25*(2), 195–199. https://doi.org/10.1007/s10164-007-0040-5

Ramírez-Restrepo, L., Koi, S., & MacGregor-Fors, I. (2017). Tales of urban conservation: *Eumaeus* butterflies and their threatened cycad hostplants. *Urban Ecosystems*, *20*(2), 375–378. https://doi.org/10.1007/s11252-016-0599-0

Robbins, R. K., Cong, Q., Zhang, J., Shen, J., Riera, J. Q., Murray, D., Busby, R. C., Faynel, C., Hallwachs, W., Janzen, D. H., & Grishin, N. V. (2021). A switch to feeding on cycads generates parallel accelerated evolution of toxin tolerance in two clades of *Eumaeus* caterpillars (Lepidoptera: Lycaenidae). *Proceedings of the National Academy of Sciences of the United States of America*, *118*(7). https://doi.org/10.1073/pnas.2018965118

Ruiz–García, N. (2020). Effectiveness of the aposematic *Eumaeus childrenae* caterpillars against invertebrate predators under field conditions. *Animal Biodiversity and Conservation*, *43*(1), 109–114. https://doi.org/10.32800/abc.2020.43.0109

Ruiz-García, N., Méndez-Pérez, B. Y., Velasco-García, M. V., Sánchez-de la Vega, G., &

Rivera-Nava, J. L. (2015). Distribución, ciclo biológico y tabla de vida de *Eumaeus toxea* (Lepidoptera: Lycaenidae) en la provincia fisiográfica Costa de Oaxaca, México. *Revista Mexicana de Biodiversidad*, *86*(4), 998–1003. https://doi.org/10.1016/j.rmb.2015.05.014

Santos Murgas, A., & Abrego, J. C. (2016). Historia Natural de *Eumaeus godartii* (Lycaenidae, Lepidoptera) y Herbivoría en Zamia manicata. *Revista Colón Ciencias, Tecnologia y Negocios*, *3*(1), 36–48.

Segalla, R., Pinheiro, F., & Morellato, L. P. C. (2021). Reproductive biology of the South American cycad *Zamia boliviana*, involving brood‐site pollination. *Plant Species Biology*, *36*(2), 348–360. https://doi.org/10.1111/1442-1984.12322

Tang, W. (1990). Reproduction in the Cycad *Zamia pumila* in a Fire-Climax Habitat: An Eight-Year Study. *Bulletin of the Torrey Botanical Club*, *117*(4), 368–374. https://doi.org/10.2307/2996834

Taylor Blake, A. S. (2020). *Eumaeus godartii* butterfly: Pest friend or foe? In C. Lopez-Gallego, M. Calonje, M. Griffith, & J. Khuraijam, *Proceedings of Cycad 2015: The 10th International Conference on Cycad Biology.*

Taylor Blake, A. S., Haynes, J., Stevenson, D., Holzman, G., & Mendieta, J. (2012). Biogeographic Insights in Central American Cycad Biology. In L. Stevens (Ed.), *Global Advances in Biogeography*. InTech. https://doi.org/10.5772/32253

Valencia-Montoya, W. A., Tuberquia, D., Guzmán, P. A., & Cardona-Duque, J. (2017). Pollination of the cycad *Zamia incognita* A. Lindstr. & Idárraga by Pharaxonotha beetles in the Magdalena Medio Valley, Colombia: A mutualism dependent on a specific pollinator and its significance for conservation. *Arthropod-Plant Interactions*, *11*(5), 717–729. https://doi.org/10.1007/s11829-017-9511-y
